# Risk factors for acute respiratory infections in children under five years attending the Bamenda Regional Hospital in Cameroon

**DOI:** 10.1186/s12890-018-0579-7

**Published:** 2018-01-16

**Authors:** Alexis A. Tazinya, Gregory E. Halle-Ekane, Lawrence T. Mbuagbaw, Martin Abanda, Julius Atashili, Marie Therese Obama

**Affiliations:** 1 Department of Internal Medicine and Pediatrics, Faculty of Health Sciences University of Buea, P. O. Box 63, Buea, Cameroon; 2 Department of Surgery and Obstetrics/Gynecology, Faculty of Health Sciences, University of Buea, P.O Box 12, Buea, Cameroon; 3 Department of Public Health and Hygiene, Faculty of Health Sciences, University of Buea, P.O Box 12, Buea, Cameroon; 4 Faculty of Medicine and Biomedical Sciences, University of Yaoundé 1, Yaoundé, Cameroon

**Keywords:** Acute, Respiratory infections, Risk factors, Proportion, Under-five

## Abstract

**Background:**

Acute respiratory infections (ARI) are a leading cause of morbidity and mortality in under-five children worldwide. About 6.6 million children less than 5 years of age die every year in the world; 95% of them in low-income countries and one third of the total deaths is due to ARI. This study aimed at determining the proportion of acute respiratory infections and the associated risk factors in children under 5 years visiting the Bamenda Regional Hospital in Cameroon.

**Methods:**

A cross-sectional analytic study involving 512 children under 5 years was carried out from December 2014 to February 2015. Participants were enrolled by a consecutive convenient sampling method. A structured questionnaire was used to collect clinical, socio-demographic and environmental data. Diagnosis of ARI was based on the revised WHO guidelines for diagnosing and management of childhood pneumonia. The data was analyzed using the statistical software EpiInfo™ version 7.

**Results:**

The proportion of ARIs was 54.7% (280/512), while that of pneumonia was 22.3% (112/512). Risk factors associated with ARI were: HIV infection OR_adj_ 2.76[1.05–7.25], poor maternal education (None or primary only) OR_adj_ 2.80 [1.85–4.35], exposure to wood smoke OR_adj_ 1.85 [1.22–2.78], passive smoking OR_adj_ 3.58 [1.45–8.84] and contact with someone who has cough OR_adj_ 3.37 [2.21–5.14].

Age, gender, immunization status, breastfeeding, nutritional status, fathers’ education, parents’ age, school attendance and overcrowding were not significantly associated with ARI.

**Conclusion:**

The proportion of ARI is high and is associated with HIV infection, poor maternal education, exposure to wood smoke, passive cigarette smoking, and contact with persons having a cough. Control programs should focus on diagnosis, treatment and prevention of ARIs.

## Background

Acute respiratory infections (ARIs) are a major cause of morbidity and mortality worldwide [1, 2]. Each year, about 1.3 million children under 5 years die from acute respiratory infections worldwide [3]. ARI constitute one third of the deaths in under five in low income countries [4]. The World Health Organization (WHO) estimates that respiratory infections account for 6% of the total global burden of disease; this is a higher percentage compared with the burden of diarrheal disease, cancer, human immunodeficiency virus (HIV) infection, ischemic heart disease or malaria [5]. Each year ARIs account for over 12 million hospital admissions in children less than 5 years [6]. In a 16 year study on the causes and circumstances of death in northern Cameroon, 67% of all deaths were in children and a majority 24% (167) of the deaths were caused by ARIs, followed by malaria 21% (152) and diarrheal diseases 19% (133) making ARIs one of the leading public health problems in under-fives in Cameroon [7].

Despite the burden of acute respiratory infection on morbidity and mortality in children under the age of five in the world, there is limited data to evaluate the problem in Cameroon. The availability of data on the proportionand risk factors of ARIs is vital because, achieving the Sustainable Development Goal on improving health and wellbeing, will depend on the existing efforts to prevent and control ARIs in all WHO regions [1, 8].

There are many socio-cultural, demographic and environmental risk factors that predispose children less than 5 years to acquire Respiratory Tract Infections (RTIs). Even though many of these risk factors are preventable [9], they have not been documented in many regions in Cameroon making it difficult to develop algorithms for the management of this group of patients.

This study therefore aimed at determining the proportion of ARIs and their associated risk factors amongst children under 5 years of age who attend the Bamenda Regional Hospital.

## Methods

### Study design and setting

This was a hospital-based cross-sectional analytic study carried out in the peak period of the dry season from December 2014 to February 2015. Bamenda is the capital of the North West Region. It is located 366 km north-west of the Cameroonian capital, Yaoundé. It has an estimated population of about 500,000 inhabitants [10] Bamenda like other parts of Cameroon has two seasons in a year. The dry season starts in November and ends by March while the rainy season starts in April and ends in October. The dry season is characterized by cool, dry and very dusty harmattan winds which blow during the peak periods of the dry season (December, January and March), with temperatures ranging between 9°c and 30°c. Many people in Bamenda use wood for cooking and this produces smoke rendering the children vulnerable to its effects.

The Bamenda Regional Hospital (BRH) is the biggest health care facility in the region receiving about 150 patients daily, about one-third being children. The hospital is a second level health facility and serves as a teaching hospital for the Faculty of Health Sciences, of the University of Bamenda. It has an HIV treatment center with over 80 children in care who visit the hospital at least monthly for a refill of their medication. This study was carried out in the pediatric unit of the Regional Hospital which has a capacity of 34 and 15 beds for pediatric patients and neonates respectively. It has a level of occupancy of more than 85% most times of the year [11]. Health care services are provided by a pediatrician, 3 general practitioners and 15 nurses. Children visiting this hospital benefit from the Expanded Program of Immunisation which offers many vaccines for various ages including the influenza vaccine, pneumococcal vaccine and the measles vaccines.

### Sample size calculation

The sample size was calculated using the formula for estimating proportions [12]. A pre-study estimate of prevalence of ARI from a systematic review of literature from 12 developing countries was 22% [13] with a precision of 0.036. The calculated sample size w as508. Five hundred and twelve children were included in the study.

### Study population and sampling

All children under 5 years who visited the Bamenda Regional Hospital during the study period and whose parents or guardians gave consent were included in the study. Children less than 2 months were excluded from this study because the clinical definitions of ARIs have low sensitivity and specificity in this age group and the clinical presentation is nonspecific. Children whose parents or guardians did not give consent were also excluded from the study.

## Study procedure

### Approach to participants

Participants were recruited between 8 am and 5 pm from Monday to Saturday from both the out-patient and inpatient departments. The parents or guardians of the child were informed about the study at the outpatient waiting room or in the wards and their written consent sought. Those who gave their consent to the study were met in the pediatrician’s consultation room or in the wards and a designed questionnaire was administered by the principal investigator. Findings from the consultation were used and additional information needed was obtained from a complementary history and physical examination.

### Data collection

Data was collected using a structured questionnaire on the; demographic, clinical and the socioeconomic variables of the child and the parents or guardians.

### Data management

Data was entered, cleaned, and analyzed using the statistical software Epi info version 7.

Case definition for ARI was was based on the Integrated Management of Childhood Illnesses (IMCI) classification for children presenting with cough or difficulty breathing.: Mild ARI (no pneumonia), Moderate ARI (pneumonia) and Severe ARI (severe pneumonia) [14]. Potential risk factors included: age, sex, birth weight, co-infection with HIV (diagnosed using a rapid test- Determine) passive smoking (any child living with someone who smokes at home), attendance at day care, exposure to wood smoke (any child who spends more than 30 min in wood smoke daily), living in the same house with someone have a cough, poor parental education (primary school level or less), malnutrition (Z-scores<2SD), inadequate immunization (if a child was lacking some vaccines for age) and mixed breastfeeding (if the child was not exclusively breastfed for at least 4 months) overcrowding (either ≥6 persons in a house, or ≥3 people using one bed) [5]. The predictor variables were grouped into three categories; socio-demographic, environmental, and clinical factors. The prevalence of ARI in children attending the BRH was calculated as; Number of children 2–59 months diagnosed with ARI during the study period, divided by the total number of children 2–59 months who visited the hospital during the same) multiplied by 100%.

The chi square test was used to evaluate significance of associations between ARI and potential risk factors, which were coded as categorical variables. The odds ratio (OR) of having ARI was calculated for all the assessed risk factors as follows: OR = (a/b)/(c/d) where a = exposed with ARI, c = not exposed with ARI, b = exposed without ARI, d = not exposed without ARI.

Multivariable analysis was performed using a logistic regression model, which included as explanatory variables all risk factors whose *p*-value was lower than 0.20 in univariable analysis.

## Results

A total of 620 children visited the hospital during the study period. Forty eight children were under 2 months of age, 36 parents did not give consent either because they presented as emergencies or did not want any delay in the consultation process. Eighteen guardians did not give consent either because they were not sure of child’s history or they did not want to enroll the children without their parents consent. Six questionnaires had incomplete information. In total, 512 children participated in the study. A majority (58%) were male and aged between 13 to 59 months (56%), with a median age of 15 months. Children older than 1 year had an average of 2.2 (SD2.4) episodes of ARIs before reaching their first birthday. Those with ARI spent a mean number of 6 (SD4.1) days with symptoms before consulting a health centre or hospital.

**Fig. 1:**
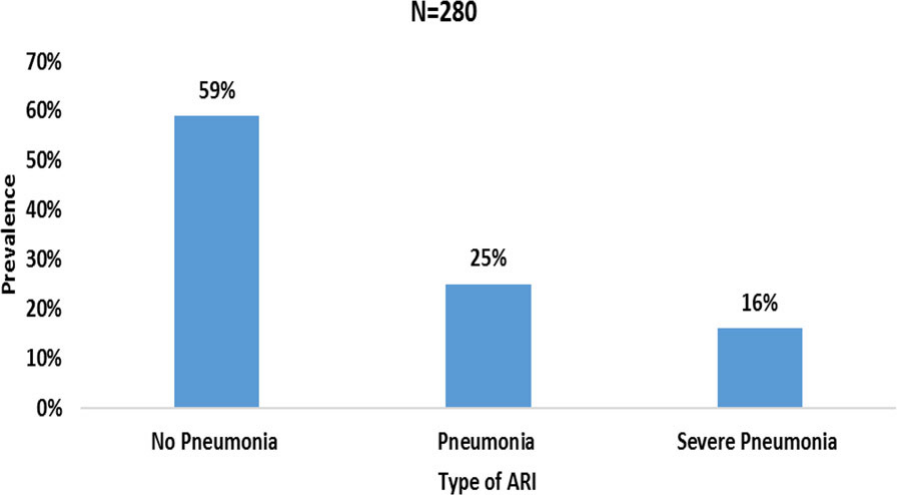
Proportion of ARI using IMCI definition in children less than 5 years

**Fig. 2:**
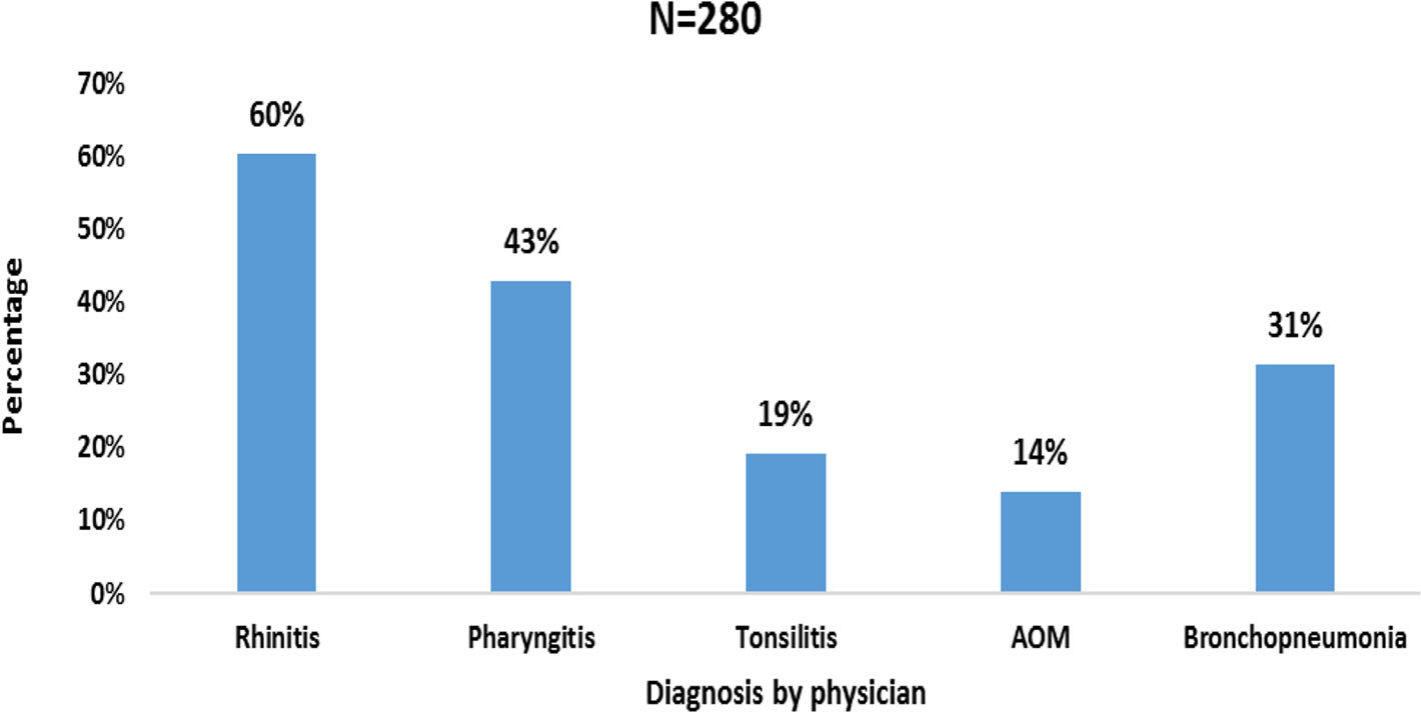
Proportion of the different types of ARI as diagnosed by physician

The proportion of ARIs in children under 5 years in the BRH was 54.7% (280 children) with a 95% CI of 50.3%–59.0%. Using IMCI guidelines, a total of 166/280 (59%) were mild ARIs (No Pneumonia), 69/280 (25%) were moderate ARI (Pneumonia) and 45/280 (16%) were severe ARI (Severe Pneumonia) as shown in Fig. 1. The ARIs as diagnosed by consultants were: rhinitis 170/280 (60%%), pharyngitis 120 (43%), tonsillitis 54 (19%), acute otitis media 39 (14%), bronchopneumonia 88 (31%) as shown in Fig. 2. One hundred and ninety one (37.3%) of the 512 children had an URTI (rhinitis, pharyngitis, tonsillitis, acute otitis media), 88 (17.2%) had a LRTI (bronchopneumonia) and 35 had both upper and lower RTI.

**Fig. 3:**
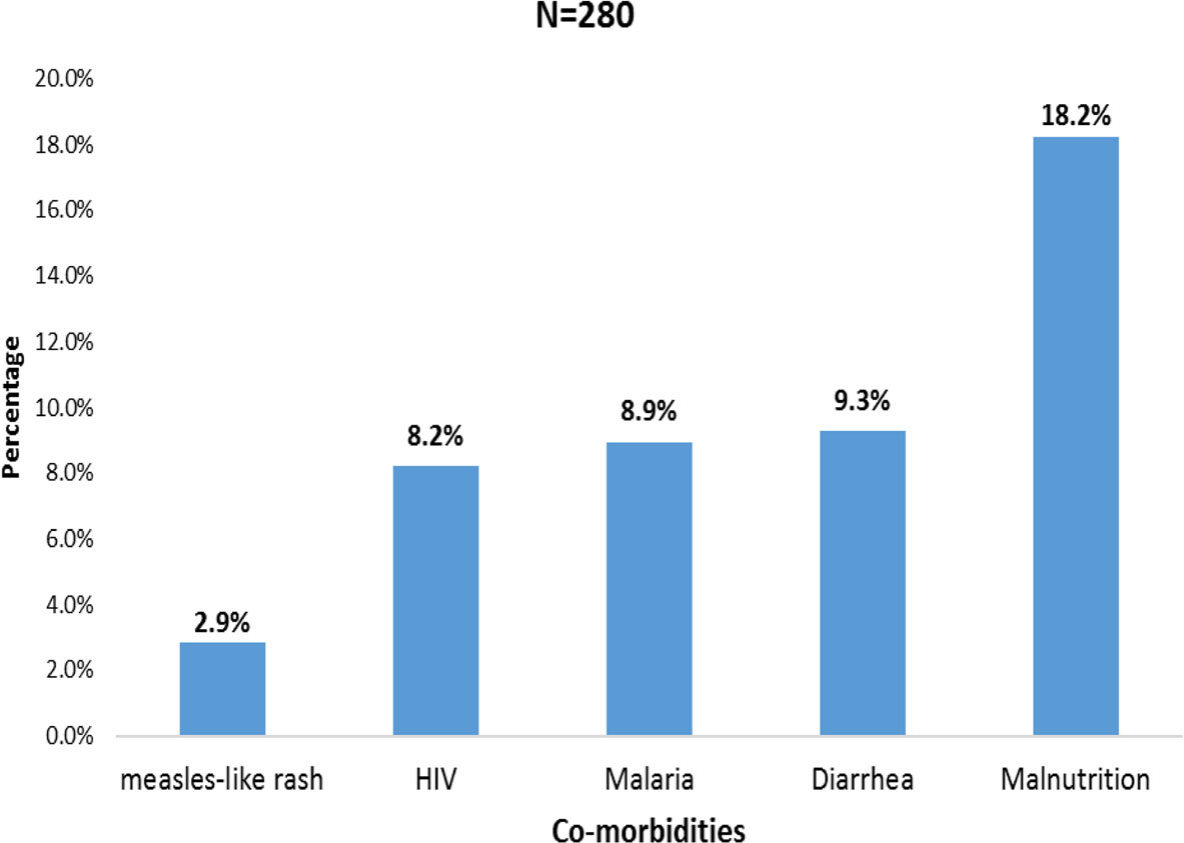
ARI and co-morbidities

Of the 280 children who had ARIs, 118 of them were also found to have other co-morbid conditions like: malnutrition 51 (18.2%), malaria 25 (8.9%), diarrheal disease 26 (9.3%), measles-like rash 8(2.9%), and HIV infection 23 (8.2%) as shown in Fig. 3.

As shown on Table 1, children aged more than 12 months, males, children with low birth weight, poor paternal education and children with younger fathers (≤30 years), accounted for a higher proportion of ARIs than their comparative groups.. However, the difference was not statistically significant (*p* > 0.05).

**Table 1:** Socio-demographic factors associated with ARI in children under 5 years

Factor	Total	ARI	Odds ratios	95% CI	*P*-value
Age(months)					
2–12	225	117(52.00%)	1		
13–59	287	163(56.79%)	1.21	0.85–1.72	0.28
Gender					
Female	218	113(51.83%)	1		
Male	294	167(56.80%)	1.21	0.85–1.72	0.29
Birth weight					
Normal	463	251(54.21%)	1		
Low birth weight < 2.5 kg	19	12(63.16%)	1.45	0.56–3.74	0.45
Overweight ≥4 kg	30	17(58.62%)	1.19	0.55–2.53	0.66
Mothers age (years)					
Age > 20	407	212(52%)	1		
Age ≤ 20	20	11(55%)	1.12	0.45–2.7	0.80
Fathers age(years)					
Age > 30	303	153(50.50%)	1		
Age ≤ 30	108	61(56.48%)	1.27	0.82–1.98	0.28
Mother’s level of education					
Secondary + tertiary	316	144(45.57%)	1		
None + primary	183	131(71.58%)	3.13	2.11–4.64	< 0.001
Father’s level of education					
Secondary + tertiary	330	173(52.42%)	1		
None + primary	159	97(61.01%)	1.42	0.97–2.09	0.07

Children with poorly educated mothers had significantly higher proportion of ARIs (*P* < 0.001) with an OR of 3.13 (95% CI: 2.11–4.64).

The evaluated clinical risk factors (Table 2) revealed that malnourished children [OR 3.01 (95% CI: 1.66–5.43)] and children infected with HIV [OR 2.88 CI: 1.21–6.83], had a significantly higher proportion (*P* < 0.05) of ARI than well-nourished and HIV negative children.

**Table 2:** Clinical factors associated with ARI

Factor	Total	ARI	Odds ratios	95% CI	*P*-value
Nutritional status					
Malnourished	67	51(76.12%)	3.01	1.66–5.43	< 0.001
Normal	445	229(51.45%)	1		
HIV status					
Positive	30	23(76.67%)	2.88	1.21–6.83	< 0.016
Negative	482	257(53.32%)	1		
Immunization status					
No/ incomplete vaccination	11	4(36.36%)	0.69	0.20–2.39	0.56
Up to date with EPI	501	227(55.29%)	1		
Breastfeeding					
Mixed	74	43(58.11%)	1.17	0.71–1.92	0.54
Exclusive	438	238(54.34%)	1		

Children who were inadequately breastfed and inadequately immunized did not have a significantly different proportion of ARIs when compared those who were exclusively breastfed and immunized respectively. (*P* > 0.05).

Environmental factors such as exposure to wood smoke, cigarette smoke, and contact or living with someone who had a cough were found to significantly increase the proportion of ARIs (P < 0.05). While the association of school attendance and overcrowding was not significant. (Table 3).

**Table 3:** Environmental and social factors associated with ARI

Factor	Total	ARI	Odds ratios	95% CI	*P*-value
Exposure to wood smoke					
Not exposed	203	82(40.39%)	1		
Exposed	309	198(64.08%)	2.63	1.83–3.79	< 0.001
Cigarette smoke					
Non passive smoker	474	249(52.53%)	1		
Passive smoker	37	31(83.78%)	4.67	1.91–11.40	< 0.001
History of contact^a^					
No contact	313	134(42.81%)	1		
Had contact	199	147(73.87%)	3.78	2.56–5.56	< 0.001
School attendance					
Do not go to school	391	210(53.71)	1		
Go to school	121	70(57.85%)	1.18	0.78–1.79	0.42
Overcrowding					
Not overcrowded	303	167(55.12%)	1		
Overcrowded	209	114(54.55%)	0.97	0.69–1.39	0.90

^a^contact with someone who has a cough

After multivariate analysis, all significant variable remained significant except for the children’s nutritional status with a *p*-value of 0.06. Table 4.

**Table 4:** Multivariate logistic regression analysis of potential risk factors for ARI

Factor	Adjusted Odds ratios	95% CI	*P*-value
Nutritional status			
Normal	1		
Malnourished	1.91	0.97–3.76	0.06
History of contact			
No	1		
Yes	3.37	2.21–5.14	< 0.01
Exposure to wood smoke			
Not exposed	1		
Exposed	1.85	1.22–2.78	< 0.01
HIV status			
Negative	1		
Positive	2.76	1.05–7.25	0.04
Mother’s level of education			
Secondary + tertiary	1		
Primary + none	2.80	1.85–4.25	< 0.01
Passive smoking			
No	1		
Yes	3.58	1.45–8.84	< 0.01

## Discussion

This study aimed at determining the proportion of ARIs and identifying some related risk factors in children under 5 years attending the Bamenda Regional Hospital. A high proportion of ARI of 54.7% was probably because this study was carried out during the peak of the dry season (December, January and February) which is characterized by dry, cold and dusty harmattan winds. Though high, this result is lower than 69.7% obtained by Sikoilia et al. in Kenya [15] and 70% obtained by Rhamam in Bangladesh [16]. This is probably because participants in this study were found to have a lower average of 3.7 ± 4.5 infections a year, compared to 6–8 episodes obtained in under-five children in Nigeria by Ujunwa et al. [4]. The number of hospital visits for ARI(2.1 ± 2.2) compared to the number episodes of ARIs in our study are similar to findings in a study in India [17] where only 42.5% of mothers regarded ARIs as serious enough to present to the hospital.

On the other hand our study found a higher proportion of ARI compared to the 10–40% in found in other studies [18]. This differences in the proportions of ARI could be as a result of different study populations, different study settings, differences in age groups studied, or because this study used mainly clinical definitions for the cases which is more sensitive than laboratory confirmed cases. A study lasting at least one complete calender year could help get the proportion of infections for a typical year. In order to reduce this high burden of ARI on the population, the ministry of public health in developing countries could include control of ARIs in their community intervention activities.

The proportion of pneumonia in this study was 22.3%, higher than the 19.4% for the Northwest Region of Cameroon as reported in 2004 by Tchatchou [19]. The proportion of a LRTI in our study (17.35%) is very similar to the 17.4% found in the Far North Region in 2011 [20] and higher than the prevalence for the Northwest Region (9.5%) in the same survey. No particular reason was found for this differences.

The proportion of pneumonia alone in this study (22.3%) is higher than the proportion of all LRTI (17.2%) in the same study. This is because the diagnostic criteria of pneumonia according to Integrated Management of Childhood Illnesses (IMCI) guidelines is highly sensitive [14] and will include some false positive cases of pneumonia, made up of children with a severe URTI because of the presence of cough, difficulty breathing with or danger signs which are sensitive but not specific to pneumonia alone. The proportion of LRTI of 17.2% in our study is similar to 19% in a study in India [21]. The diagnostic challenges of respiratory illnesses in our setting compels many clinicians to use the IMCI which does not specify the different types of ARIs but uses the term pneumonia to facilitate management in resource poor settings. Community health workers should be trained on the use of IMCI guidelines so that they can recognize ARI early enough and take appropriate actions to prevent its spread and severity.

Of the risk factors identified in our study, malnutrition was found to be significant with an odds ratio of 3.01 (95% CI: 1.66–5.43). This finding is consistent with a similar study in Nigeria by Ujunwa et al. in 2014 [4] where malnutrition was a significant risk factor with a relative risk of 3.33 (95% CI: 2.65–4.21) and Rahman in Bangladesh who obtained the prevalence of ARI in malnourished children to be 63.1% (*p* < 0.001). After multivariate analysis, the nutrition status was only marginally significant (*p* = 0.06) this could be attributed to statistical methods because the influence of malnutrition on ARIs is well known from many different studies [18, 21–25]. Supplementation of common foods for children and routine education of mothers during vaccination clinics could help address the problem of malnutrition and reduce the impact of ARIs.

The prevalence of HIV infection in Cameroon stood at 5.5% by 2007 with 420,000 new infections in children under 15 years [26]. Children who tested positive for HIV in this study (30) were more likely to have an ARI than those who were negative (OR 2.88 95% CI:1.21–6.83). Although 14 (45%) of the 31 children who tested positive for the HIV rapid test (Alere Determine ™ HIV- 1/2) were exposed chidren, the association of HIV and ARI is expected because HIV infection is known to weaken the immune system and increase the spectrum of organisms that infect the respiratory system [27]. Children who tested positive for the rapid HIV test had a higher risk and a poorer outcome than the HIV negative children [27, 28]. The presence of the HIV treatment center in the hospital can account for the considerably large number of HIV exposed and infected children. The diagnosis of HIV using rapid test will include exposed noninfected children accounting for a large proportion (8.2) of children diagnosed with HIV.

Children from mothers who had no education or primary education only, had a higher chance of developing an ARI than children from more educated mothers (secondary education and above). This is probably because children spend more time with their mothers, and mothers’ educational level will determine the quality of care and many social and environmental factors that the child will be exposed to. Ujunwa et al. also found an association with maternal education but it was significant only for LRTI and not for URTI. Other studies have also found a positive association between poor maternal education and ARI [4, 16–18, 21, 29]. Infant welfare clinics could be used as an area to reinforce maternal health education and care of infants and children.

The odds of developing an ARI after exposure to wood smoke was 2.63 compared to those who were not exposed. WHO reports that children who are exposed to cooking fuels increase the risk of developing pneumonia [1]. A similar association between wood fuel and ARI has also been found to be significant in some studies [15, 18, 21, 24, 30, 31]. The community has to be educated on the dangers of wood smoke especially because it is the main source of cooking fuel in the local communities.

Passive cigarette smoking from this study, was found to be a significant risk factor of ARI increasing the odds by 4.67 (1.91–11.40) compared to children who were not passive smokers. This is a consistent finding with other studies [4, 18, 24, 25] in which the risk of passive smoking increased by about 2 to 4 fold that of non-passive smokers [32]. This association was expected and could be explained by the fact that smoking destroys the natural protective mechanism of the respiratory tract making it easier for pathogens to overcome the first line defense of the respiratory system [33]. Anti smoking campaigns could help sensitise the population on the dangers of tobacco smoke in general and on the health of children in particular.

Coming in contact with someone who had symptoms of respiratory disease significantly increases the risk of a child to develop an ARI. This result only confirms the fact that ARIs are communicable diseases transmitted by droplets from infected persons. This is an association that has been found in other studies like Ariane et al. in the Netherlands [29]. Children should be kept away from people who present with cough so as to prevent them from getting infected.

The age, and gender of a child in this study did not significantly affect the proportions of acute respiratory infections. The meta-analysis by Jackson et al. reported an inconsistency in the effect of age and gender on ARI as 4 of the studies reviewed found a significant association and 3 others studies agree with our finding in that they did not also find a significant association of the age and gender with ARI [30].

The WHO in a report on pneumonia, brings out the importance of low birth weight in the prevalence of ARIs [24]. We did not find any significant association between the birth weight and ARIs in our study. Similarly, Lira et al., in Brazil did not find any significant difference between low birth weight and the prevalence of cough [34]. This is probably because the effect of low birth weight on ARI is more significant in neonates and our study excluded neonates.

Factors like inadequate breastfeeding and poor immunization in our study, are in great contrast with the results from many other studies [4, 18, 21, 23, 29, 30, 32]. Many breastfed children are given water and other liquids foods like corn soup frequently before 4 months of life, classifying them as mixed feeding in this study. Giving clean water and corn soup to children less than 4 months may not increase their risk of infection. Children in Bamenda receive the pneumococcal and *Haemophilus influenza* type b vaccines at 6 weeks, 10 weeks and 14 weeks and at 6 months they receive the miseasles vaccine as required by the EPI calendar. Our study did not find a significant association between vaccination and ARI probably because some of the inadequately vaccinated children according to the definition, might have received some doses of the pneumococcal and *Haemophilus influenza* type b vaccine which are known to reduce morbidity and mortality from ARI [1]. Analyzing nasal swabs could also determing the specific predisposing germs.

## Study limitations

The diagnosis of the various ARIs was made based on clinical findings and compared to the gold standards is less sensitive and specific.

This study is a hospital-based study and less than 50% of children with ARIs go to the hospital for medical care so the proportion may not be a true reflection of what is in the community.

A longitudinal study would better illustrate the effects of the potential risk factors than this cross sectional study.

## Conclusion

The proportion of ARI in the BRH was 54.7% and that of pneumonia was 22.3%. The risk factors significantly associated with ARI were: infection with HIV, poor maternal education, passive smoking, exposure to wood smoke and contact with person having ARI. Measures taken to abate these conditions will reduce the morbidity and mortality associated with ARI.

## Abbreviations

ARI: Acute Respiratory Infection
BRH: Bamenda Regional Hospital
CI: Confidence Interval
EPI: Expanded Program of Immunization
HIV: Human Immunodeficiency Virus
IMCI: Integrated Management of Childhood Illnesses
LRTI: Lower Respiratory Tract Infection
OR: Odds Ratio
RTI: Respiratory Tract Infection
SD: Standard Deviation
URTI: Upper Respiratory Tract Infection
WHO: World Health Organization

## References

[1] WHO (2006). Pneumonia, the forgotten killer of children.

[2] The UN Inter-agency Group for Child Mortality Estimation . Levels & Trends in Child Mortality: Report 2014. New York: UNICEF; 2014.

[3] Organization WH, UNICEF. Ending preventable child deaths from pneumonia and diarrhoea by 2025: the integrated global action plan for pneumonia and diarrhoea (GAPPD). 2013. 10.1136/archdischild-2013-305429 25613963

[4] Ujunwa F, Ezeonu C (2014). Risk factors for acute respiratory tract infections in under-five children in Enugu Southeast Nigeria. Ann Med Health Sci Res.

[5] Mizgerd JP (2006). Lung infection--a public health priority. PLoS Med.

[6] Nair H, Simoes EA, Rudan I, Gessner BD, Azziz-Baumgartner E, Zhang JS (2013). Global and regional burden of hospital admissions for severe acute lower respiratory infections in young children in 2010: a systematic analysis. Lancet (London, England).

[7] Einterz EM, Bates M (2011). Causes and circumstances of death in a district hospital in northern Cameroon, 1993-2009. Rural Remote Health.

[8] Griggs D, Stafford-Smith M, Gaffney O, Rockstrom J, Ohman MC, Shyamsundar P, et al. Policy: Sustainable development goals for people and planet. Nature. 2013;495(7441):305–7. 10.1038/495305a 23518546

[9] Schluger NW, Koppaka R (2014). Lung disease in a global context. A call for public health action. Ann Am Thorac Soc.

[10] Council BC (2015). Bamenda City profile.

[11] Suzuki M, Thiem VD, Yanai H, Matsubayashi T, Yoshida LM, Tho LH (2009). Association of environmental tobacco smoking exposure with an increased risk of hospital admissions for pneumonia in children under 5 years of age in Vietnam. Thorax.

[12] Wechsler S. Statistics at Square One. Ninth Edition, revised by M. J. Campbell, T. D. V. Swinscow, BMJ Publ. Group, London, 1996. No. of pages: 140. ISBN 0-7279-0916-9. Statistics in Medicine. 1997;16(22):2629-30.

[13] Selwyn BJ (1990). The epidemiology of acute respiratory tract infection in young children: comparison of findings from several developing countries. Coordinated Data Group of BOSTID Researchers. Rev Infect Dis.

[14] Patwari AK, Raina N (2002). Integrated Management of Childhood Illness (IMCI): a robust strategy. Indian J Pediatr.

[15] Sikolia DN, Mwololo K,HC. The prevalence of acute respiratory infections and the associated risk factors: a study of children under five years of age in Kibera Lindi village, Nairobi, Kenya. J Natl Inst Public Health. 2002;51(1):32-38

[16] Rahman MM, Shahidullah M (2001). Risk factors for acute respiratory infections among the slum infants of Dhaka city. Bangladesh Med Res Counc Bull.

[17] Debasism B, Ahemed T (2013). Study of knowledge, attitude and practice among mothers towards acute respiratory infection in urban and rural communitiesof burdwan district, West Bengal, India. Rev Progr.

[18] Arifeen S, Black RE, Antelman G, Baqui A, Caulfield L, Becker S (2001). Exclusive breastfeeding reduces acute respiratory infection and diarrhea deaths among infants in Dhaka slums. Pediatrics.

[19] Tchatchou ND. Variations Regionales de la Survenance des Infections Respiratoires Aigues chez les Enfants de Moins de Cinq Ans Au Cameroun: Maters[Dissertation]Yaounde, Universite de Yaounde II; 2011.

[20] INS I. Enquête Démographique et de Santé et à Indicateurs Multiples du Cameroun 2011. Calverton, MD: International I. 2012;423.

[21] Mathew JL, Patwari AK, Gupta P, Shah D, Gera T, Gogia S (2011). Acute respiratory infection and pneumonia in India: a systematic review of literature for advocacy and action: UNICEF-PHFI series on newborn and child health, India. Indian Pediatr.

[22] Mtango FD, Neuvians D, Korte R. Magnitude, presentation, management and outcome of acute respiratory infections in children under the age of five in hospitals and rural health centres in Tanzania. Trop Med Parasitol. 1989;40(2):97-102. 2772524

[23] Bellos A, Mulholland K, O'Brien KL, Qazi SA, Gayer M, Checchi F. The burden of acute respiratory infections in crisis-affected populations: a systematic review. Confl Health. 2010;4:3. 10.1186/1752-1505-4-3 PMC2829474 20181220

[24] WHO. Programme for the control of acute respiratory infections. Acute respiratory infections in children: case management in small hospitals in developing countries A manual for doctors and other senior health workers. Geneva: World Health Organization; 1990.

[25] Rahman M, Rahman A. Prevalence of acute respiratory tract infection and its risk factors in under five children. Age. 1997;23(2):47-50 9465435

[26] Mbanya D (2008). Current status of HIV/AIDS in Cameroon: how effective are control strategies?. Int J Environ Res Public Health.

[27] Zar HJ (2004). Pneumonia in HIV-infected and HIV-uninfected children in developing countries: epidemiology, clinical features, and management. Curr Opin Pulm Med.

[28] Madhi SA, Petersen K, Madhi A, Khoosal M, Klugman KP. Increased disease burden and antibiotic resistance of bacteria causing severe community-acquired lower respiratory tract infections in human immunodeficiency virus type 1-infected children. Clin Infect Dis. 2000;31(1):170–6. 10.1086/313925 10913417

[29] Macedo SE, Menezes AM, Albernaz E, Post P, Knorst M (2007). Risk factors for acute respiratory disease hospitalization in children under one year of age. Rev Saude Publica.

[30] Jackson S, Mathews KH, Pulanic D, Falconer R, Rudan I, Campbell H (2013). Risk factors for severe acute lower respiratory infections in children: a systematic review and meta-analysis. Croat Med J.

[31] Morris K, Morganlander M, Coulehan JL, Gahagen S, Arena VC (1990). WOod-burning stoves and lower respiratory tract infection in american indian children. Am J Dis Children.

[32] Arcavi L, Benowitz NL (2004). CIgarette smoking and infection. Arch Intern Med.

[33] Valencia-Gattas M, Conner GE, Fregien NL (2016). Gefitinib, an EGFR tyrosine kinase inhibitor, prevents smoke-mediated ciliated airway epithelial cell loss and promotes their recovery. PLoS One.

[34] Lira PI, Ashworth A, Morris SS (1996). Low birth weight and morbidity from diarrhea and respiratory infection in northeast Brazil. J Pediatr.

